# Extragonadal germ cell tumor of the posterior mediastinum in a child complicated with spinal cord compression: a case report

**DOI:** 10.1186/s12887-018-1070-6

**Published:** 2018-03-05

**Authors:** Dong Keon Yon, Tae Keun Ahn, Dong Eun Shin, Gwang Il Kim, Moon Kyu Kim

**Affiliations:** 1 Department of Pediatrics, CHA Bundang Medical Center, CHA University School of Medicine, Seongnam, Republic of Korea; 2 Department of Orthopedic Surgery, CHA Bundang Medical Center, CHA University School of Medicine, Seongnam, Republic of Korea; 3 Department of Pathology, CHA Bundang Medical Center, CHA University School of Medicine, Seongnam, Republic of Korea; 4 Department of Pediatrics, Severance Hospital, Yonsei University College of Medicine, Seoul, Republic of Korea

**Keywords:** Germinoma, Spinal cord compression, Mediastinal neoplasms, Case report

## Abstract

**Background:**

Germ cell tumors (GCTs) in children are rare neoplasms with diverse pathological findings according to the site and age of presentation. The most common symptoms in children with mediastinal GCTs, which are nonspecific, are dyspnea, chest pain, cough, hemoptysis, vena cava occlusion syndrome, and fatigue/weakness. Because of these nonspecific symptoms, it is difficult to suspect a mediastinal mass. A posterior mediastinal tumor causing spinal cord compression is an important example of an oncologic emergency arising from a neurogenic tumor.

**Case presentation:**

Children with posterior mediastinum GCTs can be easily mistaken as having a neurogenic tumor because of site of tumor origin. We treated our 7-year-old patient with emergency decompression surgery and high-dose steroid pulse therapy to prevent secondary injury to the spinal cord. Primary injury was a result of spinal cord compression due to the initial manifestation of GCT in the posterior mediastinum. Cisplatin-based chemotherapy was also administered. The patient was followed up regularly for 3 years and is undergoing rehabilitation without any signs of recurrence.

**Conclusions:**

We present an extremely rare case of a child with paraparesis caused by extradural spinal cord compression as the initial manifestation of GCT in the posterior mediastinum. The child was treated with emergency decompression surgery and high-dose pulse steroid therapy to prevent secondary injury to the spinal cord.

## Background

Germ cell tumors (GCTs) in children are rare neoplasms with diverse pathological findings according to the site and age of presentation. Pediatric GCTs predominantly occur in the midline of the trunk: in the intracranium, mediastinum, gonads, and sacrococcygeum. Histologic subtypes of mediastinal GCTs in children include mature teratoma, 60%; mixed GCTs, 20%; and embryonal carcinoma, 20% (including seminoma/germinoma, immature teratoma, yolk sac tumor, and choriocarcinoma). About 5% of all extragonadal germ cell tumors originate from the mediastinum in children younger than 15 years [1, 2].

The most common symptoms of mediastinal GCTs in children are dyspnea, chest pain, cough, hemoptysis, vena cava occlusion syndrome, and fatigue/weakness [3, 4]. Although survival rates of pediatric GCTs have improved significantly to 80% among patients who receive platinum-based chemotherapy, detection of mediastinal GCTs is challenging because of these nonspecific symptoms. In this article, we report an extremely rare pediatric case of paraparesis caused by extradural spinal cord compression as the initial manifestation of GCT in the posterior mediastinum. This case illustrates the importance of promptness and treatment method, and the prognosis of spinal cord compression caused by GCTs.

## Case presentation

We report the case of a 7-year-old boy, who was healthy until the age of 6 years when he presented to a local hospital with progressive intermittent back pain (grade 4 on the Numeric Rating Scale), leg weakness, and poor appetite. Within a month, he was admitted to our pediatric emergency department because he could no longer stand without assistance. At that time, he had no bladder and bowel dysfunction and no history of trauma. On neurological examination, he had significant lower extremity weakness, with a grade 3 in both legs on the Medical Research Council (MRC) scale and with preservation of sensory function. Moreover, he had positive Babinski reflexes in his lower extremities and the presence of ankle clonus. We planned an emergency imaging examination, suspecting spinal cord compression syndrome.

**Fig. 1:**
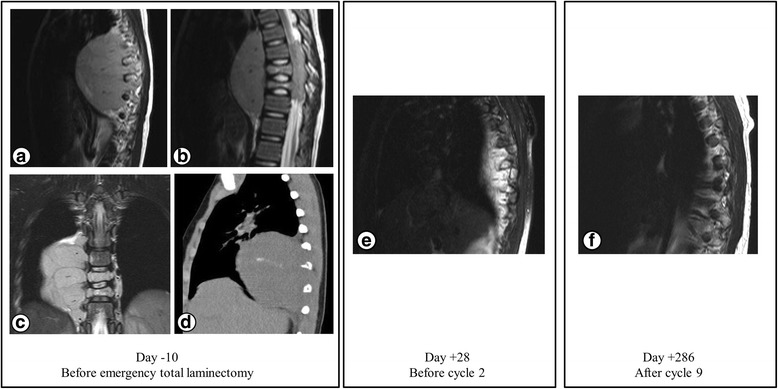
**a** Sagittal T1 and **b** T2-weighted MRI scans of the thoracic spine reveal a large mass sized 6.1 × 7.4 × 9.5 cm in the right posterior mediastinum from T5-T11, involving the T8 and T9 vertebral body with extension into the spinal canal and compressing the spinal cord. The tumor had a vertebra plana appearance and shows mixed osteolytic and sclerotic bone destruction. **c** The tumor shows septal contrast enhancement on the coronal T2 view and **d** internal dystrophic calcification on CT of the chest. **e** On day + 28 (before the 2nd cycle of chemotherapy), a thoracic spine MRI shows a much-decreased soft tissue tumor in the right posterior mediastinum at T5-T11 level with a residual lesion after removal in the spinal canal from the lower T5 -T9 level with total laminectomy from T6-T10 vertebra. **f** On day + 286 (after the 9th cycle of chemotherapy), a sagittal T2-weighted MRI on follow-up shows that the soft tissue tumor had almost disappeared from the right posterior mediastinal and intercostal space compared with the previous MRIs

Magnetic resonance imaging (MRI) of the thoracic spine and computed tomography (CT) of the chest revealed a large mass sized 6.1 × 7.4 × 9.5 cm in the right posterior mediastinum from T5 to T11, involving T8 and the T9 vertebral body with extension into the spinal canal and compressing the spinal cord (Fig. 1a, b). Moreover, the tumor showed septal contrast enhancement on coronal T2 view of the thoracic spine MRI and internal dystrophic calcification on the chest CT (Fig. 1c, d). No metastasis was observed on examination with bone scintigraphy, positron emission tomography–CT, and testicular sonography.

**Fig. 2:**
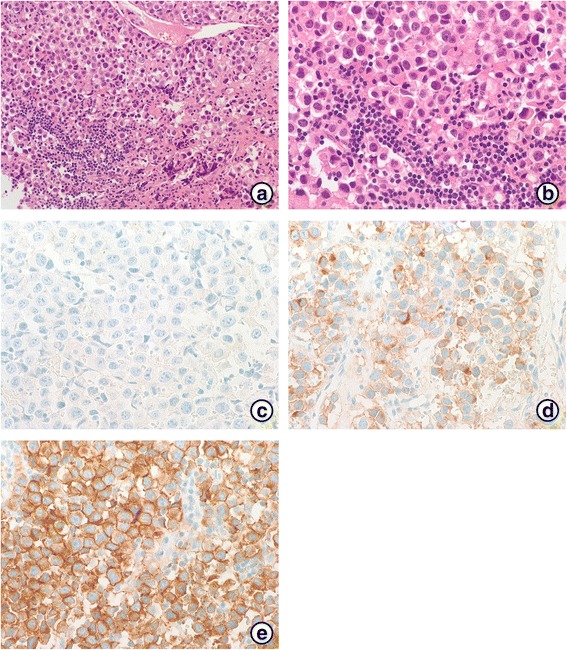
**a** Photomicrograph of the surgical specimen showing that the tumor consisted of uniform cells divided into clusters by fine fibrous trabeculae associated with a lymphocytic infiltrate (H&E, 200X). **b** Round or polygonal seminoma cells with a distinct membrane (H&E, 400X). **c** Immunohistochemical staining with β-HCG. The tumor cells were negative for β-HCG (β-HCG, 400X). **d** Seminoma stained with PALP (PALP, 400X). **e** c-KIT showed diffuse positivity in the malignant cells. (c-KIT, 400X)

Emergency total laminectomy for T6-T10 was performed and histopathological examination of the specimen confirmed a seminoma in the posterior mediastinum. Immunohistochemical staining result was negative for β-human chorionic gonadotropin (HCG), but positive for placental alkaline phosphatase (PALP) and c-KIT (CD117) in the tumor cells (Fig. 2).

**Fig. 3:**
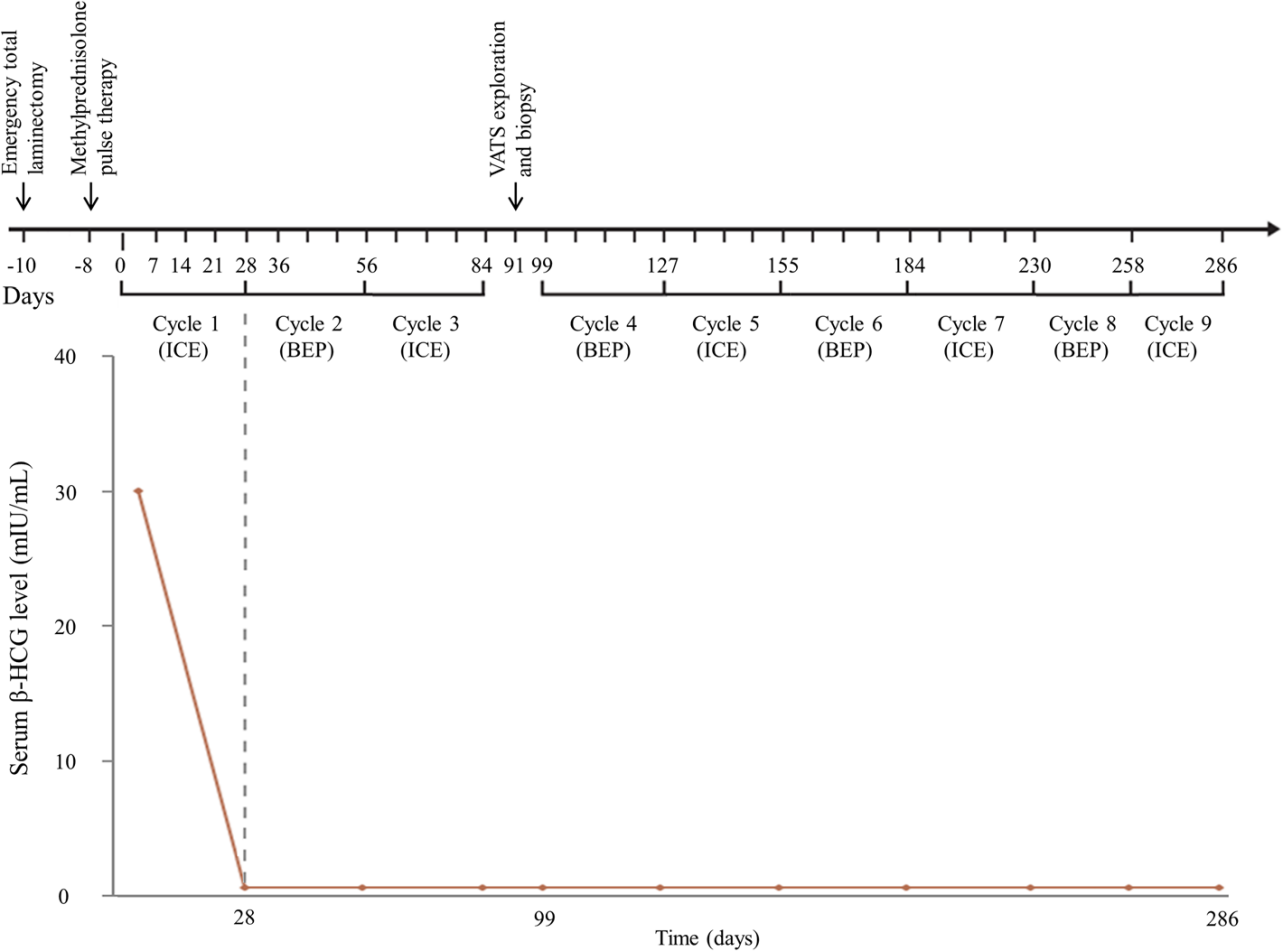
The timeline of treatment including chemotherapy, surgery, and serum β-HCG concentration. We administered methylprednisolone pulse therapy on day − 8 and alternative ICE and BEP chemotherapy. Moreover, video-assisted thoracoscopic exploration and biopsy were performed on day + 91. In the serum, β-HCG level was 30.84 mIU/mL before cycle 1. β-HCG level was evaluated in every chemotherapy cycle and was undetectable in the blood after cycle 2, as expected

Complete blood cell count and an admission panel test revealed no abnormal findings, including levels of lactate dehydrogenase 547 U/L, α-fetoprotein (AFP) < 1.3 ng/mL, and alkaline phosphatase (ALP) 172 U/L (PALP portion was not found). However, β-HCG level was high (30.84 mIU/mL, reference range 0–10 mIU/mL). The patient was treated with methylprednisolone pulse therapy (30 mg/kg/day) postoperatively for 30 h, subsequently followed by maintenance therapy with oral prednisolone for 2 weeks, as well as rehabilitation. Nine cycles of chemotherapy were administered postoperatively, followed by 5 cycles of ICE (Ifosfamide 1500 mg/m^2^/day IV on days 1-5, Carboplatin 150 mg/m^2^/day IV on days 1-2, Etoposide 100 mg/m^2^/day IV on days 1-5) and 4 cycles of BEP (Bleomycin 15 mg/m^2^/day IV on day 1, Etoposide 100 mg/m^2^/day IV on days 1-5, Cisplatin 20 mg/m^2^/day IV on days 1-5) alternatively (Fig. 3).

On day 28 (+ 28) after starting chemotherapy, before the 2nd cycle of chemotherapy, β-HCG was undetectable in the blood. Moreover, a thoracic spine MRI showed a much decreased soft tissue tumor in the right posterior mediastinum at T5-T11 level with a residual lesion after removal in the spinal canal from lower T5-T9 level, with the total laminectomy from the T6-T10 vertebra (Fig. 1e).

He underwent video-assisted thoracoscopic exploration and biopsy on day + 91 (after the 3rd cycle of chemotherapy). A grossly visible tumor was not detected and the tissue specimen was free of tumor microscopically.

After the last (ninth) cycle of chemotherapy (on day + 286), a spine MRI revealed an improved state of compressive myelopathy at the T7-T9 region and that the soft tissue tumor had almost disappeared from the posterior mediastinal and intercostal space compared with the initial MRI (Fig. 1f).

The patient has been undergoing regular physical therapy and regular follow ups for 3 years since the chemotherapy ended. On day + 1129, there was no abnormality except for a mild kyphotic curvature on the spine MRI. Moreover, the patient slowly recovered from the neurologic symptoms, improving to grade 4 in lower extremity strength on the MRC scale, and could walk without assistance. He has been wearing a thoracolumbosacral orthosis brace for kyphotic curvature prevention and is undergoing multidisciplinary rehabilitation.

## Discussion and conclusion

Spinal cord compression requires accurate diagnosis and rapid treatment to prevent progression to irreversible nerve injury. The most common etiologies of spinal cord compression are lumbar disc herniation, spinal stenosis, and masses including primary and metastatic tumor. The warning signs of back pain identified as spinal cord compression show profound unexplained neurological deficits such as motor or sensory weakness in the lower extremity, recent bowel or bladder dysfunction, and suggest non-musculoskeletal disorders [5]. Additionally, it may be helpful to examine the Babinski sign, presence of clonus, and increased deep tendon reflexes to make a diagnosis.

If the spine is in an unstable condition due to direct compression, which may result in edema, venous congestion, and demyelination, immediate decompression surgery is required for remyelination and recovery of neurologic function [6]. After prolonged compression of the spine and occurrence of spinal cord infarction, it is not possible to achieve a meaningful recovery. However, the optimal treatment for spinal cord compression caused by a primary tumor in children is unclear in a non-emergency condition. The risk of spinal deformity after multi-modality treatment must be considered carefully, especially in younger children [7]. Particularly, radiation therapy for spinal cord compression in younger children was carefully considered because of the potentially irreversible complications of radiation therapy such as spinal deformity, neurological sequelae, and secondary malignant diseases like radiation-induced lymphoma.

Steroid administration after direct decompression surgery aims to prevent secondary injury caused by posttraumatic spinal ischemia, and to improve neurologic function and spinal cord blood flow [8]. The key mechanism in the theory of secondary injury is posttraumatic ischemia in the spinal cord with resultant infarction followed by alterations in microvascular perfusion, inflammation, lipid peroxidation, free radical generation, apoptotic/necrotic cell death, and dysregulation of ionic homeostasis [9]. Based on this theory of secondary injury after direct decompression surgery, the patient received steroid pulse therapy and maintenance therapy at a physiologic dose. In a previous report, preoperative spinal angiography in pediatric inferior posterior mediastinal tumors was used to prevent secondary spinal cord ischemia due to guiding surgical resection [10].

The most common primary malignancies in the posterior mediastinum in children are neurogenic tumors, accounting for 89% of cases, as reported previously, with neuroblastoma being the most common of these [11, 12]. The child with posterior mediastinum seminoma can be easily mistaken as having a neurogenic tumor because of the symptoms and location of tumor origin. Radiographically, these seminomas appear as large, heterogeneous, lobulated soft-tissue masses with areas of hemorrhage and necrosis, and coarse, mottled, ring-shaped calcifications described in approximately 50% of thoracic neuroblastoma [13]. Mediastinal seminomas in children are typically large, homogeneous, lobulated, and well-marginated masses in the anterior mediastinum, but calcification is distinctly uncommon. However, malignant non-seminomatous GCTs differ from those with irregular margins including yolk sac tumor, choriocarcinoma, embryonal carcinoma, and immature teratoma [14, 15].

In this patient, the serum β-HCG level was increased but immunohistochemical staining after total laminectomy was negative for β-HCG. This discrepancy might have been caused by the limited tissue specimen obtained from the emergency surgery for the spinal cord compression, which was not obtained from the main mass. After the surgery, further specimens were unavailable because of the favorable response to chemotherapy.

Survival rates of pediatric GCTs have dramatically improved to more than 80% after the introduction of platinum-based chemotherapy [16]. Current platinum-based chemotherapy protocols are selected based on age, site of origin, histology of tumor, stage, completeness of surgical resection, and treatment response monitored by tumor markers such as AFP and β-HCG, and via imaging studies [17]. The measurement of tumor markers such as AFP and β-HCG helps to ascertain treatment response and determine diagnostic and therapeutic strategies.

An age exceeding 12 years is the most important factor indicative of poor prognosis of event-free survival for extragonadal malignant GCTs in children treated with cisplatin-based therapy. Elevated AFP and advanced tumor stage are also factors indicative of poor prognosis [18, 19]. In this case of a GCT, as the child was 7 years old with a normal serum AFP level and no evidence of metastasis, a good outcome is expected.

Spinal cord compression due to a posterior mediastinal tumor is considered an oncologic emergency and is mostly caused by neurogenic tumors. However, we report an extremely rare case of GCTs not arising from a neurogenic tumor, such as neuroblastoma. We treated the patient with emergency decompression surgery and high-dose steroid pulse therapy to prevent secondary injury to the spinal cord. An ICE/BEP alternative chemotherapy based on cisplatin was administered. He has been followed up regularly for 3 years and is undergoing rehabilitation without any signs recurrence.

## Abbreviations

AFP: α-fetoprotein
ALP: Alkaline phosphatase
BEP: Bleomycin, Etoposide, and Cisplatin
CT: Computed tomography
GCTs: Germ cell tumors
HCG: Human chorionic gonadotropin
ICE: Ifosfamide, Carboplatin, and Etoposide
MRC: Medical Research Council
MRI: Magnetic resonance imaging
PALP: Placental alkaline phosphatase
VATS: Video-assisted thoracoscopic surgery
